# Metastatic Crohn’s Disease in External Genitalia With Good Outcome on Adalimumab: A Rare Case of a Saudi Female and a Short Review

**DOI:** 10.7759/cureus.43380

**Published:** 2023-08-12

**Authors:** Hend M Alotaibi, Amany A Fathaddin, Hanadi M AlMutairi, Maha M Barakeh

**Affiliations:** 1 Dermatology, King Saud University, Riyadh, SAU; 2 Pathology, King Saud University, Riyadh, SAU; 3 Medicine, King Saud University, Riyadh, SAU

**Keywords:** case report, granuloma, non-caseating, metastatic, cutaneous, crohn’s disease

## Abstract

Crohn’s disease (CD), an inflammatory bowel disease that involves the gastrointestinal tract, is observed in daily hospital practice. On the other hand, metastatic Crohn's disease (MCD) is a rare entity in which cutaneous lesions are found in regions apart from the digestive system. This article describes a rare case of cutaneous CD in a Saudi female, which manifested initially as vulvar and perianal skin lesions. The diagnosis was proven by skin biopsy, and adalimumab offered effective treatment. Although cutaneous MCD is rare, it is an important cutaneous manifestation, as early detection creates the possibility of accessing effective management.

## Introduction

Metastatic Crohn’s disease (MCD) is defined by the formation of cutaneous granulomas that are not associated with the digestive system in Crohn's disease (CD) patients, represented by histology as a non-caseating granuloma [1]. MCD lesions can appear anywhere on the skin, have different morphologies, and manifest throughout all age ranges in both sexes. They may occur before, develop alongside, or occur after gastrointestinal involvement [2].

Here, we report a rare incidence of cutaneous CD in a Saudi female who had not been previously diagnosed with CD and was not experiencing gastrointestinal symptoms. The patient first presented with vulvar and perianal skin lesions. Subsequently, the diagnosis was proven by skin biopsy and consent was obtained from the patient.

## Case presentation

**Figure 1:**
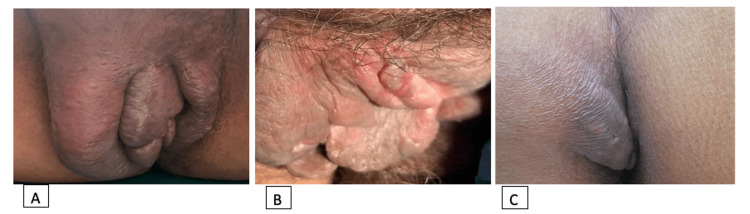
(A) Unilateral enlarged and swollen erythematous to violaceous labia majora and minora. (B) Well-defined skin-colored to erythematous perianal nodule with erosions and ulcers. (C) Skin-colored subcutaneous nodules.

A 48-year-old woman, known to have epilepsy on levetiracetam, phenytoin, and lamotrigine, and bilateral mesial temporal sclerosis, presented anxiously to the dermatology clinic for vulvar and perianal skin lesions for three years. The lesions were painful in association with swollen and red labia majora. She had a history of recurrent abscesses over the lips and was treated with a 10-day course of amoxicillin-clavulanic acid (Augmentin). She denied having any gastrointestinal diseases or a family history of similar conditions. There was no history of weight loss, fever, or appetite loss. Physical examination revealed unilateral enlarged and swollen erythematous to violaceous labia majora and minora with well-defined skin-colored to erythematous perianal nodules without ulcers, as well as bilateral inguinal lymphadenopathy (Figure 1).

**Figure 2:**
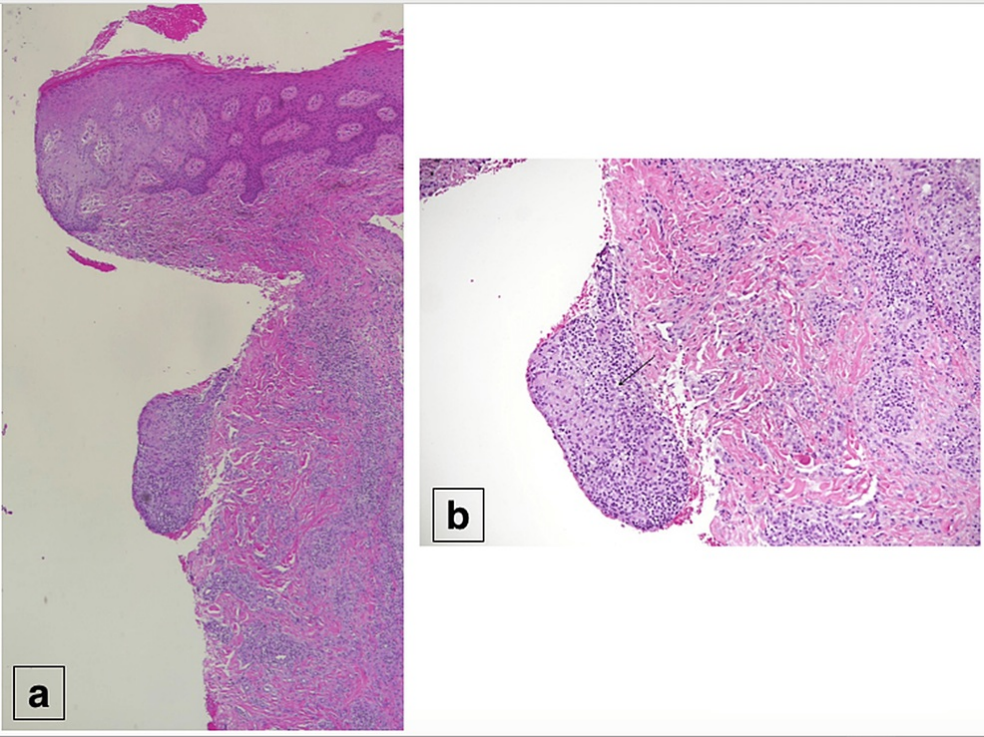
The microscopic section of the skin biopsy showing a hyperplastic epidermis with non-necrotizing granuloma in the dermis. (a) Hematoxylin and eosin (H&E), x4. (b) H&E, x100.

The microscopic section of the hematoxylin and eosin (H&E)-stained tissue sections from the peri-anal skin punch biopsy revealed a hyperplastic epidermis. In the dermis, there was a focus of non-necrotizing granuloma with adjacent mixed dermal inflammation (Figure 2).

Magnetic resonance imaging (MRI) of the pelvis was done and showed marked cutaneous thickening and induration of subcutaneous fat of the pubis, perineum, and labia on the left side. The patient was examined by the gastrointestinal team. Results for blood inflammatory markers were unremarkable. Colonoscopy was performed, which was normal up to the terminal ileum (TI). Multiple specimens for histopathological examination were taken from the ascending colon, transverse colon, descending colon, and rectum. Except for the ascending colon specimen, which showed no significant pathology, all biopsies showed mild to moderate chronic inactive inflammation. Crypt morphology in colonic mucosa showed mild lymphoplasmacytosis, hyperplastic lymphoid follicles, and hypertrophic muscularis mucosa. No polyp, ulcer, activity (cryptitis), granuloma, viral cytopathic change, dysplasia, or invasive carcinoma was noted.

**Figure 3:**
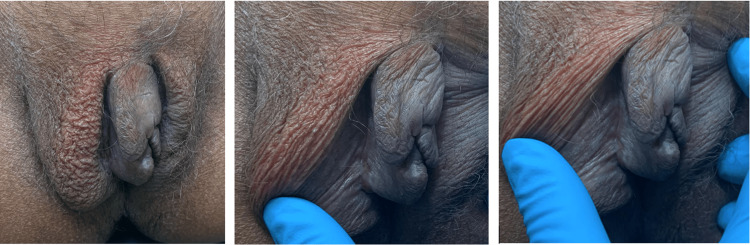
After three months from starting adalimumab. The pictures demonstrate a reduction in redness and labial swelling.

Initially, the patient was started on doxycycline 100 mg oral (PO) daily with worsening and progression of the lesions. Then, a trial of metronidazole 200 mg orally three times a day for one month resulted in a slight improvement. After all, the patient's condition improved significantly after receiving adalimumab 160 mg subcutaneously as the loading dose, reducing it to 80 mg, then 40 mg subcutaneous injections every two weeks with good outcomes. Three months after starting adalimumab, the patient responded well to treatment in the form of less swelling size, pain, and itchiness (Figure 3). The patient is still taking adalimumab and is satisfied with the outcome.

## Discussion

MCD can occur months to years before the onset of gastrointestinal symptoms, more frequently in children [3]. The underlying pathogenesis is unknown, yet it has been hypothesized that in primary CD, immune complexes or gastrointestinal antigens circulate across the body and deposit in the skin, producing the perivascular granulomatous characteristics visible in microscopic analysis of MCD lesions [4]. Both genders are affected; however, females are more commonly observed [5]. Prevalence and incidence are not well-documented in the literature due to the limited available data and are estimated to be overlooked due to the varying clinical appearance [6].

MCD, known as a great mimicker, presents polymorphic clinical characteristics that are similar to other dermatoses. Skin lesions can appear as nodules, erythematous to purple plaques, and ulcers. The vulva, penis, trunk, and face are the next most often affected areas after the limbs, especially the legs. Areas that were flexural and intertriginous were likewise susceptible. On rare occasions, multiple or disseminated lesions are visible [7]. Clinically, MCD may resemble other conditions, such as erysipelas, cellulitis, or hidradenitis suppurativa [1]. When considering a differential diagnosis for MCD, it is important to consider several possibilities. Granulomatous disorders include tuberculosis, cutaneous sarcoidosis, syphilis, mycobacterial infections, actinomycosis, fungal infections, lymphogranuloma venereum, and granuloma inguinale. On the other hand, non-granulomatous disorders include hidradenitis suppurativa, erysipelas, cellulitis, pyoderma gangrenosum, impetigo, erythema nodosum, factitial dermatitis from factitial injection of foreign substances, schistosomiasis, chronic lymphedema resulting from obstruction, and foreign body reaction [2]. A comprehensive evaluation, including medical history, physical examination, imaging studies, laboratory tests, and a biopsy along with periodic acid-Schiff (PAS) staining, acid-fast bacilli (AFB), and tissue cultures, is necessary to reach an accurate diagnosis and differentiate MCD from these potential causes. MCD is characterized under the microscope by a non-caseating granulomatous infiltration located in the superficial papillary and deep reticular dermis with occasional extension into the subcuticular fat [4].

Due to a scarcity of randomized control trials, approaches to therapy for MCD remain inconsistent [2]. Different treatment modalities have been used with successful responses for MCD in the literature, including a variety of chemotherapeutic agents and surgical procedures combined with oral zinc sulfate [8-13]. Two reported cases of MCD involving the external genitalia had complete recovery on treatment with oral and topical steroids along with azathioprine [9,10]. Further, a recent case of MCD involving the genital area and Wolman disease was reported to have clinical improvement beginning with a 14-day treatment of topical miconazole and hydrocortisone ointment used twice daily, followed by 0.1% topical tacrolimus ointment applied twice daily [13]. Since there are no established guidelines for the treatment of MCD, the drugs we tried include doxycycline 100 mg PO daily for four weeks with worsening of the lesions. Afterward, metronidazole 200 mg orally three times a day for one month with mild improvement was started. Nonetheless, the patient’s condition improved significantly after starting adalimumab 160 mg subcutaneously as the loading dose and then tapered to 80 mg, followed by a 40 mg subcutaneous injection every two weeks. By the period of three months, signs of clinical improvement were noted. In light of the literature, adalimumab has been reported to work effectively for MCD patients [14-17]. In one case of cutaneous CD, significant improvement of skin lesions was observed after starting adalimumab 160 mg, 80 mg after two weeks, and 40 mg every other week. Remission was obtained after four months [14]. A similar situation was reported previously, which utilized the dosage of adalimumab for CD, with 160 mg administered as four injections over the first two days, 80 mg on day 15, and then 40 mg every other week following that. Six weeks after starting treatment, the subcutaneous lesions had completely resolved. Clinical remission was obtained after 20 weeks [15]. Whereas in another case of cutaneous Crohn’s presenting as genital warts, adalimumab treatment began with an 80 mg loading dose and continued with 40 mg every other week. The case described remarkable improvement after the second week, as the size of the perianal skin tags gradually reduced, and the cutaneous perianal lesions' erythema and induration subsided [16]. Furthermore, adalimumab was observed to provide favorable outcomes within six weeks when administered as 40 mg induction and maintenance injections every other week, and by six months, both cutaneous and endoscopic improvement was demonstrated [17].

The prognosis for these cutaneous symptoms resembles that of CD, and the mortality of patients with childhood-onset inflammatory bowel diseases is increased compared with the general population [8,18]. Because the hallmarks of MCD are not specific, along with the fact that 40% of CD patients have at least one extra-intestinal symptom most frequently in the skin [8], various inflammatory skin disorders can complicate CD, and MCD can be masked by other cutaneous manifestations of CD. In addition, cutaneous manifestation could precede gastrointestinal symptoms; therefore, health physicians need to be aware and take that into account early. Better knowledge and early detection of CD extraintestinal symptoms including MCD can prompt timely diagnosis for successful therapeutic intervention.

## Conclusions

MCD can be the first presentation and can precede gastrointestinal symptoms of CD. To rule out further conditions, a thorough clinical examination is helpful, along with a skin biopsy that should be done to confirm the diagnosis. Adalimumab proved successful in our situation, adding to the body of literature that has reported different therapeutic approaches. To validate the effectiveness of the therapy, further research with larger sample sizes and controlled studies is needed. Since most patients are concerned about their diagnosis and treatment possibilities, a multidisciplinary team must refine their plan to enhance patients' outcomes.
